# Lower limb clinical osteoarthritis and its association with joint pain and function, and severe injuries and surgeries, in women professional football players: a cross-sectional observational study

**DOI:** 10.17159/2078-516X/2025/v37i1a21272

**Published:** 2025-09-15

**Authors:** DA Ramagole, DC Janse van Rensburg, P Bennett, C Cowie, M Maas, R Mehta, G Ramkilawon, J Rantanen, J Salo, BM Pluim, G Kerkhoffs, V Gouttebarge

**Affiliations:** 1 Amsterdam UMC location University of Amsterdam, Department of Orthopedic Surgery and Sports Medicine, Meibergdreef 9, Amsterdam, the Netherlands; 2 Section Sports Medicine, Faculty of Health Sciences, University of Pretoria, Pretoria, South Africa; 3 UKSI Performance Centre, Loughborough University, Loughborough, UK Sports Institute; 4 The Football Association (England), National Football Centre, St George’s Park, Needwood, DE13 4PD, UK; 5 Amsterdam UMC location University of Amsterdam, Department of Musculoskeletal Radiology, Meibergdreef 9, Amsterdam, The Netherlands; 6 Academic Center for Evidence-based Sports Medicine (ACES), Amsterdam, the Netherlands; 7 Amsterdam Movement Sciences, Aging & Vitality, Musculoskeletal Health, Sports, Amsterdam, The Netherlands; 8 Department of Statistics, Faculty of Natural and Agricultural Sciences, University of Pretoria, Turku, Finland; 9 Orthopaedics and Sports Clinic, Mehilainen NEO Hospital, Turku, Finland; 10 Hospital Mehilainen, Helsinki, Finland; 11 Amsterdam Collaboration on Health & Safety in Sports (ACHSS), IOC Research Center of Excellence, Amsterdam, the Netherlands; 12 Football Players Worldwide (FIFPRO), Hoofddorp, the Netherlands

**Keywords:** clinical osteoarthritis, female, football, lower limbs, injuries, surgeries

## Abstract

**Background:**

Athletes may sustain severe injuries and need to undergo surgery, potentially leading to osteoarthritis (OA). Early diagnosis and rehabilitation can significantly improve outcomes and extend an athlete’s career.

**Objectives:**

To determine: 1) prevalence of clinical hip, knee, and ankle OA; 2) association with pain and function; 3) association with severe injuries and surgeries in female professional footballers.

**Methods:**

This cross-sectional study involved female professional footballers who completed online questionnaires on joint pain and function, severe injuries, and surgeries. Their physicians evaluated them for clinical OA (cOA) of the hips, knees, and ankles.

**Results:**

Among 74 participants, cOA prevalence of the hips, knees, and ankles was 2.7% (95% CI: 0–6.4), 5.0% (95% CI: 2.5–10.6), and 8.0% (95% CI: 1.9–14.3), respectively. Pain was present in the hips (p=0.615; Cramer’s V=0.132), knees (p=0.556; Cramer’s V=0.171), and ankles (p=0.028; Cramer’s V=0.391). Joint function was impaired in the hips (p=0.379; Cramer’s V=0.214), knees (p=0.738; Cramer’s V=0.103), and worse in the ankles (p=0.0778; Cramer’s V=0.255). A weak association existed between hip cOA and injuries (p=0.230; Cramer’s V=0.193), with moderate associations in the knees (p=0.024; Cramer’s V=0.290) and ankles (p=0.168; Cramer’s V=0.206). Surgeries were associated with knee cOA (p=0.0578; Cramer’s V=0.258) and not ankles (p=1.00; Cramer’s V=0.027).

**Conclusion:**

The prevalence of clinical OA was low among female footballers. Pain was the primary symptom, accompanied by impaired joint function. Severe knee injuries and surgeries were linked to cOA. Further research is recommended in this population.

The health benefits of exercise are well-documented. However, athletes have a higher risk of developing osteoarthritis (OA) compared to the general population,^[1]^ and there is a link with previous injuries.^[2–3]^ It has been reported that in current and retired male football players, every additional severe knee injury and additional knee surgery increases the likelihood of knee OA twofold,^[4]^ and injuries are a major contributor to the premature career end.^[5]^

The incidence of ligament injuries in the lower limbs was reportedly higher in professional women football players than in domestic-level players.^[6]^ Knee OA was higher in former women football players with a history of anterior cruciate ligament (ACL) and meniscus injuries. Both chondral and meniscal loss were significant predictors of the development of OA.^[7]^

Amongst retired German women football players, severe injuries are strongly associated with joint pain and confirmed knee and ankle OA^[8]^ with radiographic changes twelve years after an ACL injury and surgery in over 60%.^[9]^

Evidence linking joint pain, severe injuries, surgeries, and clinical OA development in female professional footballers remains limited. Among active male football players, the odds of developing knee OA increased 1.49 times with injuries and 4.35 times with surgeries. Pain was the main symptom, and joint function was impaired.^[3]^

The objectives of this study were threefold. Firstly, to establish the prevalence of clinical OA of the hips, knees and ankles. Secondly, to determine the association between joint pain and function with clinical OA of the hips, knees and ankles. Thirdly, to determine the association between severe injuries and surgeries with clinical OA of the hips, knees, and ankles among women professional football players.

## Methods

### Study designs and ethical considerations

A cross-sectional observational study utilising a questionnaire and the Strengthening the Reporting of Observational Studies in Epidemiology (STROBE) statement^[10]^ was used to ensure the reporting quality. The Medical Ethics Review Committee of the Amsterdam University Medical Centers approved this study (Drake Football Study: NL69852.018.19| W19_171#B202169). The study was conducted in accordance with the Declaration of Helsinki (2013).

### Participant selection

The International Federation of Professional Footballers (FIFPRO) and affiliated international unions recruited women professional footballers. This population is the same group used in a broader health surveillance program, which includes gynaecological health patterns and experiences of motherhood.^[11]^ Inclusion criteria were: (a) being a professional footballer, (b) a woman, (c) able to read and comprehend texts in English or French. The definition of a professional footballer was that she (i) trains to improve performance, (ii) competes in the highest or second-highest national league, and (iii) has football training and competition as a major activity or focus of personal interest, devoting several hours in all or most of the days for these activities, and exceeding the time allocated to other types of professional or leisure activities.

Sample size was calculated using the formula $n=\frac{Z^{2}\;P(1,-,P)}{d^{2}}$ where *n =* sample size, *Z =* statistic for a level of confidence (1.96 for 95% confidence level*), P =* Expected prevalence or proportion, and d = Precision. This indicated that at least 40 participants were needed to reach a power of 80% (confidence interval (CI) of 95% and absolute precision of 7% under the assumption of an anticipated population proportion (prevalence) of 5%.^[12]^

### Data collection

Physicians diagnosed cOA of the hips, knees, and ankles based on history and clinical examination. The NICE age-adapted criteria were used based on (i) activity-related joint pain, (ii) restricted range of motion of the joint, and (iii) either no morning joint-related stiffness or morning stiffness lasting no longer than 30 minutes.^[13]^ Pain in the hips, knees and ankles was assessed using three questions (e.g., ‘How often do you experience knee pain?’). This was scored on a 5-point scale ranging from 0 “Never” to 4 “Always”.

The Hip dysfunction and Osteoarthritis Outcome Score Short Form (HOOS-PS)^[14]^ was used to assess the level of hip function. Participants rated the degree of difficulty they experienced the previous week due to hip pain: descending stairs, getting in/out of a bath/shower, running, sitting, and twisting. These items were measured on a 5-point scale (from 0 to 4) and subsequently converted using the conversion Table. The total score, ranging from 0 to 100, was calculated, where 0 represents total hip disability and 100 represents perfect hip function.^[15]^

The Knee injury and Osteoarthritis Outcome Score Short Form (KOOS-PS)^[15]^ was used to assess knee function. Participants rated the degree of difficulty (ranging from ‘none’ to ‘extreme’) they experienced the previous week due to knee pain: rising from bed, putting on socks/stockings, rising from sitting, bending to the floor, twisting, kneeling, and squatting. The 7 items were measured on a 5-point scale (ranging from 0 to 4) and subsequently converted using the conversion table. The total score, ranging from 0 to 100, was calculated, where 0 represents total knee disability and 100 represents perfect knee function.

The American Academy of Orthopaedic Surgeons (AAOS)^[16]^ foot and ankle module from The Lower Limb Core Scale was used to assess foot and ankle function. This 25-item questionnaire is subdivided into five subscales: pain, other symptoms, interference with activities of daily living, interference with sports and recreation, and quality of life related to ankle and foot problems. These were each measured on a 5- or 6-point scale and entered into a computerised AAOS spreadsheet. A total score ranging from 0 to 100 was calculated, where higher scores indicated better foot and ankle function.^[16]^ These questionnaires have been validated in several study populations and languages, including English and French.^[16]^ The scores for all three questionnaires were reported as ‘mean ± standard deviation (SD)’.

The history of severe hip, knee or ankle injuries and associated surgeries during a career as a professional footballer was obtained through twelve questions (e.g., “How many severe injuries have you had in your left hip so far as a professional footballer?” and “How many surgeries in your left hip have you had so far as a professional footballer?” Severe injury was defined as an injury sustained during football activities that resulted in either training or match absence for more than 28 days.^[17]^ This information was gathered from participants or their medical professionals.

FIFPRO disseminated information about the study via email to potential participants. Interested participants gave informed consent and were asked to complete an electronic questionnaire (CastorEDC, CIWIT B.V, Amsterdam, the Netherlands), which included all dependent and independent variables and several descriptive variables (e.g., age, height, field position). Additional information on self-reported global physical and mental health was collected using the Patient-Reported Outcomes Measurement Information System Global Health form (PROMIS-GH).^[18]^

### Data analysis

Statistical analysis was performed using R Software (version 4.1.3; http://www.r-project.org).

Descriptive analyses (mean, standard deviation (SD), frequency, and range) of the participants were performed for all variables. For the study’s first objective, point prevalence was calculated to evaluate the prevalence of clinical hip, knee and ankle OA using a 95% Wald-adjusted confidence interval (CI). This was expressed as a percentage and calculated as the frequency of participants with cOA relative to the total number of participants clinically evaluated for OA. For the second and third objectives, Fisher’s Exact test was used to determine the statistical significance of the association between joint pain, function, severe injuries and surgeries with cOA. Furthermore, odds ratios (OR) were calculated to report and quantify the odds of developing cOA based on severe injuries and related surgeries. For objectives 2 and 3, a “p-value” of less than 0.05 indicated a statistically significant association, while the strength of association was calculated using the Cramer’s V metric (“0” indicative of “no strength of association” and “1” of “perfect association”). Cramer’s V value categories are calculated according to the effect size (ES) as follows:

ES ≤ 0.2 indicates a weak association, 0.2 < ES ≤ 0.6 indicates a moderate association and ES > 0.6 indicates a strong association between two values.^[19]^

## Results

### Participants characteristics

Seventy-four women athletes were recruited, with a mean age of 25.0 years (95% CI: 24.3, 25.6 years and a median (IQR) of 25.0 (23.0, 27.0 years). The mean BMI was 22.1 (95% CI: 21.8, 22.5 kg/m^2^) and a median (IQR) of 22.0 (21.1, 23.1 kg/m^2^). The number of years as a professional player was 5.7 (95% CI: 5.0, 6.5 years). Table 1 depicts demographics, football characteristics, career level, and employment status. The majority of participants were defenders (n=25; 34%), followed by forwards (n=22; 30%), midfielders (n=17; 23%), and goalkeepers (n=10; 14%).

### Prevalence of clinical OA

The prevalence of cOA was 2.7% (95% CI: 0–6.4), 5.0% (95% CI: 2.5–10.6), and 8.0% (95% CI: 1.9–14.3) in the hips, knees, and ankles, respectively.

### Clinical OA and joint pain and function

The association between cOA joint pain and function is presented in Table 2.

Among the 74 participants, 16 (22%) reported hip pain. Two (12.5%) were clinically diagnosed with hip OA. The HOOPS-PS score was higher in the OA group (13.5±19.0) than in those without OA (2.64± 6.69), indicating more disability. There was no significant association between clinical hip OA and hip pain (p=0.615; Cramer’s V value=0.132), but there was a weak to moderate association with joint function (p=0.379; Cramer’s V value=0.214).

Forty (54%) participants experienced knee pain. Four (10%) were diagnosed with clinical knee OA. The KOOS-PS score was higher in the OA group (14.7±12.58), indicating more disability than the group without OA (9.71±12.98). There was no significant association between clinical knee OA and pain (p=0.5556; Cramer’s V value=0.171) and joint function (p=0.738; Cramer’s V value=0.103).

Forty (54%) participants experienced ankle pain. Six (15%) were clinically diagnosed with ankle OA. The AAOS score was lower in the OA group (84.9±7.2), indicating more disability than the group without clinical ankle OA (95.2±6.0). There was a significant association between clinical ankle OA and ankle pain (p=0.028; Cramer’s V value=0.391), and between clinical ankle OA and joint function (p=0.078; Cramer’s V value=0.255).

### Clinical OA and severe injuries and surgeries

The association between cOA and severe injuries and surgeries, and the odds ratios are presented in Table 3.

Nine participants reported severe hip injuries, and one (11%) had cOA. None of the participants with clinical hip OA underwent any associated surgery. There was a weak association between clinical hip OA and severe injuries (p=0.230; Cramer’s V value=0.193) or surgery (p=1.0; Cramer’s V value=0.027).

Thirty participants reported severe knee injuries and four (13%) had cOA. Twenty participants underwent knee surgery, and three (15%) had cOA. There was a significant and moderate association between clinical knee OA and severe injuries (p=0.024; Cramer’s V value=0.290) and surgeries (p=0.058; Cramer’s V value=0.258).

Fifty participants reported severe ankle injuries and six (12%) had clinical ankle OA. Ten participants underwent ankle surgery, and one (10%) had cOA. There was a weak association between clinical ankle OA and severe injuries (p=0.168; Cramer’s V value=0.206) and none with surgeries (p=1.0; Cramer’s V value=0.0274).

The odds of a player developing knee OA after severe injuries were 7.6 times higher (p=0.230; Cramer’s V=0.193). This increased to 9 times higher with surgeries (p=0.058; Cramer’s V value=0.258) than in players without severe injuries or surgeries. However the odds of developing cOA in the ankles were only 1.3 times higher in those with one or more ankle surgeries than in players without ankle surgeries (p-value=1.0; Cramer’s V value=0.027).

## Discussion

Our findings were 1) a low prevalence of cOA in the hips 2.7%, knees 5.4% and ankles 8.1% in women professional football players; 2) pain was the primary presenting symptom, with joint function impairment; 3) severe injuries and surgeries were associated with knee cOA, but not with ankle cOA. This study focused on injuries and surgeries, not other contributory factors.

### Prevalence of clinical OA

Several studies have shown that retired male football players have a high incidence of hip or knee OA.^[4]^ A recent publication reported that almost 50% of retired elite women football players have radiographic evidence of knee OA before the age of 50 years.^[7]^ Our participants have a low prevalence of cOA, possibly due to the following: i) young mean age of 25 years; ii) low mean BMI of 22; iii) access to medical and surgical care; iv) physical activity.

### Clinical hip, knee and ankle OA and its association with pain and function

Pain is the primary presenting symptom in OA and has been used to describe the burden of OA as a chronic disease, often leading to poor quality of life (QoL).^[20]^ Our study confirmed that pain was the primary presenting symptom, and function was impaired.

The International Foot and Ankle Consortium (2022) reviewed evidence available for diagnosis, epidemiology, burden, outcome assessment and treatment of foot and ankle OA.^[21]^ They confirmed that there was no definition for foot and ankle OA, unlike for hip and knee OA. Health-related quality of life (HRQoL) was noted to be poorer in individuals with ankle OA, including difficulties with climbing stairs, deficiencies in gait, instability and altered pressure on the sole.^[21]^ Our study aligns with these findings, with the ankles being the most frequently injured joints.

### Clinical hip, knee and ankle OA and its association with severe injuries and surgeries

Severe injuries and associated surgeries increase the likelihood of developing knee^[4]^ and ankle^[22]^ OA in retired football players, with every additional severe injury and surgery increasing the risk.^[4]^ Our study aligns with these previous studies.

### Practical implications of the study

Clinical OA can be diagnosed early using the age-adapted NICE criteria during the career of women football players. Early diagnosis of clinical OA will enable the implementation of interventions to delay the progression of this debilitating condition, affecting athletes’ quality of life (QoL). Prevention of injuries is paramount in sports, and tools such as the FIFA 11+ Injury Prevention tool^[23]^ should be considered to minimise the burden of severe injuries, surgeries, and early development of OA.

### A post-hoc analysis of QoL

The self-reported physical and mental PROMIS-GH scores showed no significant differences between the groups with and without cOA regarding physical and mental scores. Therefore, we can conclude that QoL was not affected by cOA in this group of active players.

### Strengths and limitations

This first study in cOA in active women professional football players explored associations with pain, function, severe injuries, and surgeries using the NICE diagnostic criteria and validated functional assessment tools.

Limitations include small sample size, possible participant recall bias, different examining physicians, and though guided by the NICE criteria, experience and expertise were not standardised. We noted the association between severe injuries and surgeries with the development of cOA, but not as a causal factor.

A ten-year follow-up surveillance is planned to investigate further associations between severe injuries, surgeries and cOA development. Our research aims to promote women’s football studies, reduce gender disparities, and enhance player health and career opportunities.

## Conclusion

Lower limb cOA prevalence in women professional footballers was low. Pain was the primary presenting symptom, with impaired joint function (especially ankles). Severe injuries and surgeries correlated with higher knee cOA. These findings align with previous studies on knee and ankle problems in former women footballers. Early cOA diagnosis using adapted NICE criteria before radiographic changes appear should prompt clinicians to implement protective measures, optimal joint protection, treatment, and rehabilitation to extend careers and delay radiographic OA onset. There is a need for standardised clinical assessment protocols to minimise inter-examiner variability.

## Figures and Tables

**Table 1:** Demographics, football characteristics, employment status and career level. Data expressed as mean ± standard deviation or n (%)

Demographics	All participants (n=74)

Age (years)	25.0±2.7
Height (cm)	168.4±5.4
Weight (kg)	62.8±5.4
BMI (kg/m^2^)	22.1±1.5
Stress fractures n (%)	11 (15%)
Low bone density n (%)	1 (1.4%)

**Other characteristics**	

Defender position	25 (34%)
Forward position	22 (30%)
Midfield position	17 (23%)
Goalkeeper	10 (13%)
Seasons as professional	5.7±3.1
Employed (yes)	22 (30%)
Highest national level	64 (87%)
Second highest national level	6 (8%)
Other level	4 (5%)

**Table 2:** Prevalence of hip, knee and ankle clinical osteoarthritis and association with joint pain and function (n=74)

Clinical hip OA						

	Without OA	With OA	%	95% CI	p-value	Cramer’s V

Prevalence		2	2.7	0 – 6.4	N/A	N/A
Joint pain	14	2	12.5	N/A	0.615	0.132
Function	2.6±6.7	13.5±19.0			0.379	0.214

**Clinical knee OA**						

Prevalence		4	5.4	2.5 – 10.6	N/A	N/A
Joint pain	36	4	10.0	N/A	0.556	0.171
Function	9.7±13.0	14.7±12.6			0.738	0.103

**Clinical ankle OA**						

Prevalence		6	8.1	1.9 – 14.3	N/A	N/A
Joint pain	34	6	15	N/A	0.028*	0.391
Function	95.2±6.0	84.9±7.2			0.078	0.255

* Indicates significance (p<0.05).

OA, osteoarthritis; %, percentage of the total group; CI, confidence interval; N/A, not applicable. Joint pain and Function are arbitrary units derived from questionnaires (see methods).

**Table 3:** Prevalence of clinical OA of the hips, knees and ankles, and association with severe injuries and surgeries (n=74)

Clinical hip OA						

	Without OA	With OA	%	Odds ratio (95% CI)	p-value	Cramer’s V

Prevalence		2	2.7	0 – 6.4	N/A	N/A
Severe injuries	8	1	11.1	7.6 (0.09–633.52)	0.221	0.193
Surgeries	2	0	0	N/A	1	0.028

**Clinical knee OA**						

Prevalence		4	5.4	2.5–10.6	N/A	N/A
Severe injuries	26	4	13.3	N/A	0.024	0.290
Surgeries	17	3	15	9.0 (0.7–498.3)	0.057	0.258

**Clinical ankle OA**						

Prevalence		6	8.1	1.89–14.33	N/A	N/A
Severe injuries	44	6	10	N/A	0.168	0.206
Surgeries	9	1	10	1.31 (0.02–13.85)	1	0.027

OA, osteoarthritis; %, percentage of the total group; CI, confidence interval; N/A, not applicable.
